# Using Next-Generation Sequencing to Disentangle the Diet and Incidence of Intestinal Parasites of Falkland Flightless Steamer Duck *Tachyeres brachypterus* and Patagonian Crested Duck *Lophonetta specularioides* Sharing a South Atlantic Island

**DOI:** 10.3390/genes14030731

**Published:** 2023-03-16

**Authors:** Juan F. Masello, Yvonne R. Schumm, Sven Griep, Petra Quillfeldt

**Affiliations:** 1 Department of Animal Ecology & Systematics, Justus Liebig University Giessen, Heinrich-Buff-Ring 26, D-35392 Giessen, Germany; 2 Institute for Bioinformatics & Systems Biology, Justus Liebig University Giessen, Heinrich-Buff-Ring 58, D-35392 Giessen, Germany

**Keywords:** Anatidae, Aves, diet composition, DNA based, Illumina sequence, molecular scatology, next-generation sequencing, non-invasive, non-metric multidimensional scaling, parasite, South Atlantic

## Abstract

Species overlapping in habitat use can cohabit depending on how they exploit resources. To understand segregation in resource use, an exhaustive knowledge of the diet is required. We aimed to disentangle the diet composition of the Falkland Flightless Steamer Duck *Tachyeres brachypterus* and the Patagonian Crested Duck *Lophonetta specularioides* sharing a coastal environment. Using DNA extracted from scats and Illumina sequencing, we generated a list of molecular operational taxonomic units. Both ducks consumed a variety of invertebrates, frequently overlapping in the taxa consumed. However, only the Falkland Flightless Steamer Ducks consumed fish, which might be indicative of dietary specialization and inter-specific segregation in the restricted space that these birds share. Moreover, the female and male Falkland Flightless Steamer Ducks consumed different fish prey, with almost one-third of the fish taxa being consumed by females only and another similar number consumed by males only. This result might suggest a case of intra-specific competition, triggering sexual segregation. Additionally, we detected parasitic Platyelminthes (Cestoda and Trematoda), with different frequencies of occurrence, probably related to the different diet compositions of the ducks. This study provides the necessary baseline for future investigations of the ecological segregation of these ducks.

## 1. Introduction

Species with similar habitat requirements can coexist within a community depending on how they use the available resources. As complete competitors cannot coexist [1,2], the niche theory predicts that animals will segregate in the n-dimensional niche hyper-volume to avoid both inter- and intra-specific competition (e.g., [3,4,5,6]). Segregation can be achieved through differences in foraging behavior [7], habitat use, morphological adaptation or dietary specialization [8,9].

Analyses of the food consumed by animal species sharing a habitat can facilitate our understanding of the mechanistic processes in a community and the different functions of the organisms in an ecosystem [10]. Dietary studies give vital insights into foraging behavior [11] and prey species occurrence and abundance [12], with implications for understanding environmental change and its impact on wildlife [13,14,15]. However, detailed knowledge on the diet of a species can be difficult to obtain, particularly for small, nocturnal, beneath-the-soil, underwater, oceanic or elusive species [10,16].

In birds, traditional diet analyses were extensively based on direct observations of feeding and microscopic examinations of feces [10,16]. High-resolution photography in seabirds has also been used to study diet (e.g., [17]). However, all visual analyses are highly labor-intensive, frequently lack resolution [10] or are often restricted to assessing chick diet or the diet of birds during the breeding season [17]. Often, invasive methods were used, including lethal sampling, induced regurgitations, the stomach flushing of live individuals, the use of neck collars on nestlings and the dissection of collected individuals [17,18,19]. A powerful, non-invasive, more accurate and less labor-intensive alternative for dietary studies are DNA-based methods. Molecular methods allow for the identification of consumed species via the characterization of the DNA present in gut or fecal samples [20,21,22]. Among different approaches, DNA barcoding uses a standardized DNA region (DNA barcode), which is PCR-amplified, and amplicons are sequenced and then compared to a reference database for identification [10]. Moreover, the development of next-generation sequencing (NGS) has allowed for the identification of prey items of several samples up to the species level in a single sequencing run while upholding the ability to trace each prey back to the sample of origin [23,24]. As a consequence, NGS is increasingly being used for diet studies [11,25,26], with feces being the least invasive and most widespread sample type [21,27,28].

DNA barcoding can additionally detect the presence of parasitic organisms, particularly those related to the digestive tract if fecal samples are used [20,29,30]. Such supplementary information may allow for more comprehensive knowledge of the ecological interactions of the investigated species [20]. For instance, parasites may affect an individual’s condition or behavior, consequently altering the way the host interacts with the environment or other species [31].

The Patagonian Shelf Large Marine Ecosystem is a hot spot for marine biodiversity [32]. Adjacent to it, the Falkland/Malvinas Islands are home to a large number of birds, including 70 seabird species and some of their largest colonies [32,33,34]. In this large archipelago, New Island (51°43′ S, 61°18′ W) holds a rich avifauna, including 39 regularly breeding species representing 65% of the total breeding species [35]. Seabirds make up the largest numbers, followed by waterfowl, song birds, birds of prey and shorebirds [35]. The Falkland/Malvinas Current creates an area of ocean water upwelling, with increased productivity and a rich marine life, just west of New Island (e.g., [36,37,38]), which provides food to the large number of birds and seals on the island.

Foraging ecology and segregation in space and time have been a particular focus of our research on the birds from New Island (e.g., [7,39,40]). Recently, the use of NGS of the prey DNA present in fecal samples provided detailed information on the prey consumed during the time the birds were tracked, making it possible to understand the ecological mechanism involved in individuals’ foraging behavior [11]. However, until now, the research concentrated on seabirds (Sphenisciformes, Procellariiformes and Pelecaniformes), with the need to expand to other taxonomical groups to understand how such a large bird community (>2 million breeding pairs of seabirds only; [41]) shares the relatively restricted space of New Island (2011 ha., 84 km of coastline). In order to achieve this, we currently expanded the focus of our research to include two species of Anatidae (Anseriformes) sharing the coastal environment of New Island: the Falkland Flightless Steamer Duck *Tachyeres brachydactyla* and the Patagonian Crested Duck *Lophonetta specularoides* (Figure S1 in the Supplementary Materials). In the present study, we aimed to investigate the diet of these species as a necessary basis to understand how they segregate in the use of the shorelines of New Island.

## 2. Materials and Methods

### 2.1. Studied Species

The Falkland Flightless Steamer Duck is a species endemic to the Falkland/Malvinas Islands, where it is widespread [42]. It is frequently found in association with kelp beds, and it is numerous in sheltered harbors, where it forages by up-ending in shallow water or by diving in deep water [42,43]. The species is dimorphic, and, thus, males and females can be easily distinguished in the field (Figures S2–S4 in the Supplementary Materials). In the south of New Island, up to 73 adult birds have been recorded foraging almost always on the eastern, more sheltered coast [35]. Mainly marine invertebrates were reported as part of its diet, with adults taking mostly lobster krill *Munida gregaria* (Decapoda), followed by Gastropoda (kelp snails, limpets and *Mytilus*), Pelecypoda, Osteichthyes, hermit crab *Pagurus comptus*, Isopoda, Amphipoda and unidentified algae [44]. The young consumed a lower variety of food items, feeding on Gastropoda, Isopoda, Amphipoda, lobster krill and unidentified algae [44].

The Patagonian Crested Duck is a common resident dabbling duck, widely distributed in the Falkland/Malvinas Islands; however, it is more common around West Falkland. The species is monomorphic, and, thus, males and females cannot be distinguished in the field (Figures S2–S4 in the Supplementary Materials). As the birds were not captured for this study, sex information for this species was not available, and only the categories ‘adults’ and ‘chicks’ were considered. On New Island, up to 69 individuals were counted on sheltered sea coasts and on the South End Pond [35] (Figures S5–S7 in the Supplementary Materials). It is most commonly seen foraging in the sea in sheltered bays, favoring areas with extensive growths of green, filamentous algae, but it also forages in ponds near the sea [44]. In summer, adults were reported to feed mostly on Isopoda and Amphipoda, as well as consuming Gastropoda, shore flies (Diptera, Helcomysidae), unidentified algae and seeds of the low bush Diddle-dee *Empetrum rubrum* [44]. Young birds consumed a higher variety of food items, including Pelecypoda, Cladocera (*Daphnia*), Coleoptera and seeds of the perennial herb Pigvine *Gunnera magellanica* (Gunneraceae) [44].

### 2.2. Sample Collection

From October 2017 to February 2019, we collected fresh scat samples from Falkland Flightless Steamer Ducks (16 adult females, 22 males and 11 chicks) and Patagonian Crested Ducks (12 adults and 4 chicks) breeding at New Island/Malvinas Islands. Both species are territorial, a trait that allowed us to observe and follow the birds closely until they defecated, and we collected the samples immediately. This allowed for a clear assignation of the samples to the individuals from which they originated. We collected the scats from rock substrates along the shoreline (North End Beach, Ship Harbour, Protector Beach, Settlement, South Harbour and South End Beach, all facing the east). To avoid external contamination, we took special care to collect the central part of the scat and not the part that was in direct contact with the substrates. We kept the scat samples cool with ice packs during fieldwork, froze them once back at the field station and transported them frozen until processed in the laboratory.

### 2.3. DNA Isolation

Of each sample, we took 180–200 µg for DNA extraction, using the entire sample (minimum: 21 µg) if less material was available. We extracted the DNA from the fecal samples using a QIAamp® DNA Stool Mini Kit (Qiagen GmbH, Düsseldorf, Germany), mostly following the protocol provided with the kit. However, to ensure proper homogenization, we added 2–3 bashing beads (ZR Bashing BeadTM 2.0 mm, Zymo Research, Irvine, CA, USA) and used the Disruptor Genie™ (Scientific Industries SI™, Bohemia, NY, USA). We also increased the incubation with Buffer AL and proteinase K from 10 to 30 min. During isolation and throughout the entire process, we included two negative extraction controls, i.e., empty vials, along with the fecal samples. We determined DNA quantity and quality using UV spectrophotometry with a NanoDrop2000c UV-Vis spectrophotometer (NanoDrop Technologies, Wilmington, DE, USA), diluting samples to 20 ng/µL if the final DNA concentration was higher than 100 ng/µL.

### 2.4. Construction of Sequencing Library

We constructed a sequencing library (NGS) by means of a PCR, followed by an indexing PCR. We first used a primer targeting Bilateralia (Table 1). The first results using this primer suggested that the birds fed on Mollusca and Osteichthyes, and for this reason, we also included specific primers targeting these two groups (Table 1). In all used primers, we attached Illumina overhang adapters (P5 for forward primers: 5′-TCGTCGGCAGCGTCAGATGTGTATAAGAGACAG-3′ and P7 for reverse primers: 5′-GTCTCGTGGGCTCGGAGATGTGTATAAGAGACAG-3′). We included in all PCR runs PCR-grade water as a negative control, negative extraction controls and positive controls. We visualized the PCR amplicons using QIAxcel Advanced (QIAGEN) high-resolution capillary gel electrophoresis. As the next step, we purified a 5 µL aliquot of the amplicon PCR by means of an Illustra™ ExoproStar 1-Step Kit for enzymatic PCR clean-up (GE Healthcare, UK), according to the protocol provided. After purification, we performed an index PCR to individually mark each PCR product with specific Illumina indices added to the P5 and P7 overhang adapters. Following this, we purified and normalized the index PCR products using a SequalPrep™ Normalization Plate Kit (Thermo Fisher Scientific, Waltham, MA, USA). Finally, we pooled 2 µL of each normalized and individually tagged sample. We sent the samples for sequencing, using 250 bp paired-end reads on a MiSeq desktop sequencer (Illumina, San Diego, CA, USA) at SEQ-IT GmbH & Co. KG, Kaiserslautern, Germany.

### 2.5. Bioinformatics Analyses of Sequences

We used the raw Illumina sequence data to produce a list of molecular operational taxonomic units (MOTUs). Bioinformatics analyses included the following steps: assessing sequence quality with FASTQC http://www.bioinformatics.babraham.ac.uk/projects/fastqc (accessed on 15 March 2023), the adapter and quality trimming of the paired-end reads with TRIMMOMATIC (minimum quality score of 20 over a sliding window of 4 bp) [48], the merging of the overlapping paired-end read pairs using FLASH [49], transforming sequence files to FASTA with the FASTX-Toolkit http://hannonlab.cshl.edu/fastx_toolkit/ (accessed on 15 March 2023) and extracting amplicons in MOTHUR [50]. We used USEARCH [51] to remove identical replicates (dereplicate; derep_fulllength), to detect and to remove chimeric sequences (uchime_denovo) and to cluster sequences into molecular operational taxonomic units (MOTUs). Using the BLASTn algorithm [52], we matched MOTU sequences to reference sequences in the National Center for Biotechnology Information (NCBI) GenBank nucleotide database, using a cut-off of 90% minimum sequence identity and a maximum e-value of 0.00001. We carried out all bioinformatics analyses using a custom workflow in GALAXY https://www.computational.bio.uni-giessen.de/galaxy (accessed on 15 March 2023) [53]. We based the taxonomic assignment on the percentage similarity of the query and the reference sequences. Since short fragments are less likely to contain reliable taxonomic information, we only retained sequences with a minimum length of 190 bp and a BLASTn assignment match greater than 98% [54,55]. We assigned MOTUs to the species level in cases when all retained hits of a MOTU with the same quality criteria (sequence identity, sequence length, e-value) corresponded to the same species; if not, we assigned the MOTU to the lowest shared taxonomic level, e.g., genus or family, as in Kleinschmidt et al. [26]. Additionally, we discarded taxa with very distant or ecologically irrelevant distribution ranges (e.g., steppes, mountains). Negative controls were included and did not show any contaminations.

### 2.6. Statistical Analyses

First, we calculated the frequency of occurrence of each MOTU [56]. We also performed non-metric multidimensional scaling (NMDS), using the function *metaMDS* in the R package VEGAN [57], to visualize differences in diet compositions. NMDS collapses information originally in multiple dimensions into two dimensions to simplify visualization and analyses. It is considered the most unconstrained ordination method in community ecology [58,59]. To investigate the agreement between the two-dimensional configuration and the original configuration, we used the function *metaMDS*, which allows for the calculation of a stress parameter. A stress < 0.05 represents an excellent agreement, <0.1 is very good, and <0.2 provides a good representation. In our models, the stress was always <0.08 (very good). Additionally, we ran a permutational multivariate analysis of variance by means of distance matrices (PERMANOVA), using the function *adonis*, and we tested for the multivariate homogeneity of group dispersions (variances) using the function *betadisper*. We applied the function *ordispider* to improve visualization, connecting each sample to the centroid (the arithmetic mean position of all the points) of the category to which it belonged. We also used the function *ordiellipse* to visually emphasize the centroid of similar categories [57].

## 3. Results

**Figure 1:**
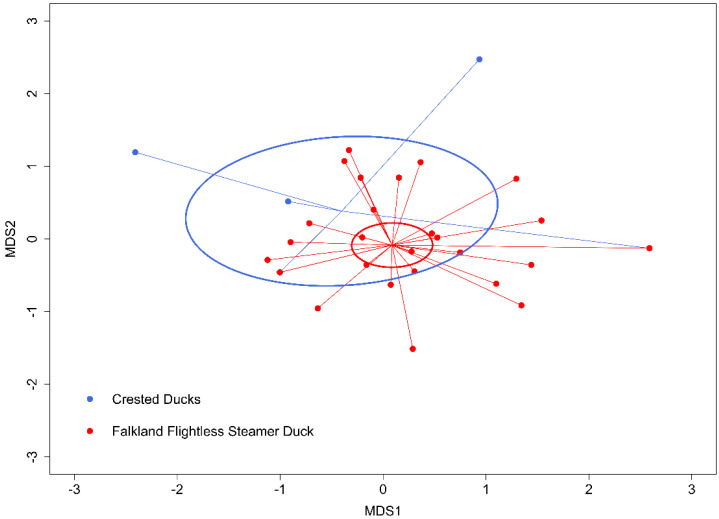
Differences in diet composition between Falkland Flightless Steamer Duck *Tachyeres brachydactyla* and Patagonian Crested Duck *Lophonetta specularoides*. Data correspond to individuals foraging on the coast of New Island, Falkland/Malvinas Islands, from October 2017 to February 2019. We used non−metric multidimensional scaling of molecular operational taxonomic units to collapse information from multiple dimensions into two dimensions to facilitate visualization and interpretation. The ellipses and lines connect similar categories.

The Falkland Flightless Steamer Ducks and Patagonian Crested Ducks frequently overlapped in the prey consumed (NMDS: *F*_38,1_ = 1.961, *P* = 0.050, where the species explained 8% of the overall variation, *R*^2^ = 0.077; Figure 1). Both species foraged on similar invertebrate prey, such as Sphaeromatidae (Isopoda), lobster krill, bivalves or Turbinidae (Gastropoda; Table 2). However, the Patagonian Crested Ducks consumed no Arachnida, Echinodermata or fish in contrast with the Falkland Flightless Steamer Ducks, which preyed upon a variety of fish (Table 3). In addition to the frequency of occurrence of the different prey taxa in Table 2 and Table 3, we also provide the diet composition using non-metric multidimensional scaling of molecular operational taxonomic units, including the family identity of the prey consumed, in Figure S8 in the Supplementary Materials.

**Figure 2:**
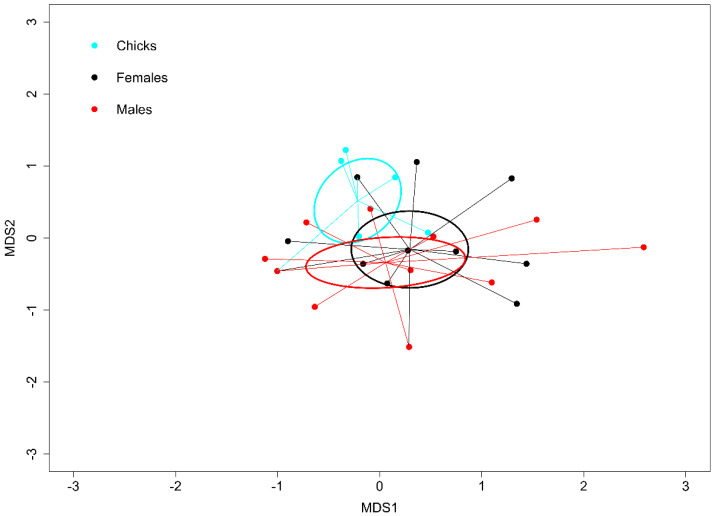
Differences in diet composition among Falkland Flightless Steamer Duck *Tachyeres brachydactyla* age and sex categories. Data correspond to individuals foraging on the coast of New Island, Falkland/Malvinas Islands, from October 2017 to February 2019. We used non−metric multidimensional scaling of molecular operational taxonomic units to collapse information from multiple dimensions into two dimensions to facilitate visualization and interpretation. The ellipses and lines connect similar categories.

When considering the chicks, females and males of Falkland Flightless Steamer Ducks separately, we found strong similarities in diet composition (NMDS: *F*_31,2_ = 1.477, *P* = 0. 092, where the categories explained 17% of the overall variation, *R*^2^ = 0.174; Figure 2). However, we detected some items exclusively in the diet of (1) females, e.g., Oribatida (Arachnida) and Molgulidae (Ascidiacea), and (2) males, namely, Holothuroidea (Echinodermata) and Fissurelloidea (Gastropoda; Table 2). Moreover, females consumed 4 of the 14 fish taxa (29%; Myctophidae, Agonidae, Eleginopsidae and Channichthyidae), which males did not consume (Table 3). Conversely, males preyed upon another four different fish taxa (Clupeidae, Engraulidae, Carangidae and Scombridae), which females did not consume (Table 3).

In addition to the diet composition, we were able to reveal the presence of parasitic Platyelminthes in the Falkland Flightless Steamer Ducks and Patagonian Crested Ducks (Table 4). We found all detected taxa but one present in the Falkland Flightless Steamer Duck samples, with particularly high frequencies of occurrence of Cestoda in chicks and of Trematoda in females (Table 4). We found fewer Platyelminthes taxa present in the samples from Patagonian Crested Ducks, of which the adult samples included only Cestoda, while those from chicks comprised both Cestoda and Trematoda (Table 4).

## 4. Discussion

DNA barcoding allowed us to gain detailed new knowledge on the diet composition and parasitic exposure of two species of Anatidae sharing the coastal environment of New Island in the southwestern Atlantic. Both the Falkland Flightless Steamer Duck and the Patagonian Crested Duck consumed a varied range of prey (Table 2 and Table 3), frequently overlapping in the taxa consumed (Figure 1). Our analyses also allowed us to detect interesting differences between the duck species. Among other prey, the Falkland Flightless Steamer Ducks preyed upon a variety of fish and a few Arachnida and Echinodermata, which were completely absent in the diet of the Patagonian Crested Ducks (Table 3). Moreover, in the only previous study investigating in detail the diet of some waterfowl species in the east of the Falkland/Malvinas Islands [44], no fish, Arachnida or Echinodermata were reported as food for the Patagonian Crested Duck, suggesting that the difference we found is a consistent pattern. This absence of fish in the diet of the Patagonian Crested Duck, in contrast to that of the Falkland Flightless Steamer Duck, could be an indication of dietary specialization, which would allow the coexistence of both Anatidae in the fairly restricted space of the New Island coast. However, to better understand the ecological segregation between both duck species, a detailed study of their foraging behavior and microhabitat use is needed. In a previous study [7], we found strong segregation among penguins and shags that allowed their coexistence on New Island. However, in addition to strong differences in the diet of those seabirds, spatial and temporal segregation during foraging were also observed [7]. In the case of those penguins and shags, we found that segregation was most probably generated by optimal foraging in relation to habitat differences on a local scale, including the distance to the coast and the bathymetric depth of the foraging areas [7]. A similar future study, using current biologging tools (e.g., [60,61,62]) in combination with further DNA-barcode-based diet analyses, would allow us to gain even more in-depth knowledge of the ecological segregation between the Falkland Flightless Steamer Duck and the Patagonian Crested Duck from New Island.

Our larger sample size of the Falkland Flightless Steamer Duck allowed us to investigate differences between the sexes. Remarkably, females and males consumed several food items, which only appeared in the diet composition of one or the other sex. Most notably, the fish consumed differed between the sexes, with almost one-third of the fish taxa being consumed by females only (Myctophidae, Agonidae, Eleginopsidae and Channichthyidae) and another almost similar number consumed by males only (Clupeidae, Engraulidae, Carangidae and Scombridae; Table 3). The reason for such differences could be intra-specific competition, triggering sexual segregation, which may take different forms, such as different prey choices between females and males, as well as spatial segregation in foraging areas and temporal segregation in foraging activities (e.g., [63,64,65,66]). In fact, this has been observed in seabirds from New Island during previous studies. In particular, Imperial Shag *Phalacrocorax (atriceps) albiventer* breeding on New Island showed strong sexual segregation in the form of diet segregation, little overlap in foraging areas and strong time segregation in foraging activities [7,67]. An additional circumstantial piece of information points to the need to further investigate the potential case of sexual segregation in the Falkland Flightless Steamer Duck. In five occasions in our current study, the samples from both members of a breeding pair were successfully sequenced. In all those five breeding pairs, one sex consumed fish, and the other did not. With our current sample size and without detailed observations of foraging behavior, we cannot rule out that either this was observed just by chance or that an ecological mechanism could be responsible. Further research taking advantage of biologging and NGS applied to the investigation of prey identity will certainly elucidate these matters.

DNA barcoding also allowed us to gain new knowledge on the parasitic exposure of the Falkland Flightless Steamer and Patagonian Crested Ducks. In the scat samples of both duck species, we were able to find DNA from parasitic worms belonging to the classes Cestoda and Trematoda (Table 4). The Falkland Flightless Steamer Ducks showed a high frequency of occurrence of Cestoda compared to the Patagonian Crested Ducks, which may be related to the absence of fish in the diet composition of the latter species. Cestoda frequently parasitize Crustacea and fish as intermediate hosts and many fish-eating mammals and birds as definitive hosts [30,68,69]. The frequency of occurrence of Cestoda in our study was similar to that in other seabirds [68] but much higher than that in seals [30], which may be related to specific diet compositions. The Falkland Flightless Steamer Ducks also exhibited a higher frequency of occurrence of Trematoda than the Patagonian Crested Ducks, with only chicks being parasitized in the latter species. Trematoda are usually transmitted through the consumption of Crustacea, Gastropoda and Bivalvia [70,71,72]. Thus, our results showing a much higher frequency of occurrence of Gastropoda and Bivalvia prey taxa in samples from the Falkland Flightless Steamer Ducks would also explain their higher prevalence of Trematoda. Such complementary results certainly allow for a more complete understanding of the ecological interactions of the studied species [20]. Parasites may strongly affect avian predators by affecting their condition or behavior, which may turn individuals more susceptible to predation and, thus, reduce their impact on an ecosystem [31,73]. They can also affect several aspects of the life history, reducing, e.g., traits such as clutch size and breeding success [74,75]. Our results on the presence of Platyelminthes provide a baseline for future studies investigating impacts on individuals and populations, e.g., their effects on the life history of the host and changes in time in the parasitic load related to environmental changes.

In our study, most prey and parasite taxa were determined at the family level, with only six taxa being assigned at the species level (Table 2, Table 3, Table 4). This was probably caused by an incomplete representation of the prey and parasite taxa in the DNA sequence reference libraries, the lack of appropriate primers or both, as suggested for other regions of the world [76,77,78]. Our current study, together with a previous one using DNA barcoding to investigate the diet of Gentoo Penguins *Pygoscelis papua* on New Island (Masello et al. 2021), provides a starting point for a much-needed taxon barcoding gap analysis. Such a study should identify the marine invertebrate taxa that need to be added to increase the barcoding coverage and, thus, enable an improved identification of fecal samples and environmental DNA in general.

## Figures and Tables

**Table 1:** List of primers used in this study for the detection of prey species and intestinal parasites in scat samples from Falkland Steamer Duck *Tachyeres brachypterus* and Crested Duck *Lophonetta specularioides*.

Prey Target	Gene	Primer Name	Sequence 5′-3′	Annealing Temperature (°C)	Amplicon Size (bp)	Reference
Bilateralia	*Nuclear 18S*	BilSSU110f	AGAGGTGAAATTSTTGGAYCG	60	∼245	[45]
		BilSSU1300r	CCTTTAAGTTTCAGCTTTGCA			
Mollusca	*16S rRNA*	L2510	CGCCTGTTTATCAAAAACAT	50	~350	[46]
		H3059	TTTCCCCGCGGTCGCCCC			
Osteichthyes	*Mitochondrial 12S*	FishF1	CGGTAAAACTCGTGCC	56	~300	[47]
		FishR1	CCGCCAAGTCCTTTGGG			

**Table 2:** Invertebrate and ascidian prey consumed by Falkland Flightless Steamer Duck *Tachyeres brachydactyla* and Patagonian Crested Duck *Lophonetta specularoides*, and frequency of occurrence (% of samples).

Phylum	Class	Order	Family	Species	Common Name	Falkland Flightless Steamer Duck			Patagonian Crested Duck	
						Females	Males	Chicks	Adults	Chicks
Annelida	Polychaeta	Sabelida	Sabellidae		feather duster worms	-	-	-	14	-
		Terebellida	Terebelldae		bristle worms	7	20	10	-	-
Arthropoda	Copepoda	Calanoida				-	-	18	43	75
			Diaptomidae			-	-	-	14	-
		Harpacticoida				-	7	-	-	-
	Branchiopoda	Anostraca			fairy shrimps	14	7	-	-	-
		Diplostraca			clam shrimps	7	27	9	-	25
			Daphniidae		water fleas	7	27	9	-	-
			Lynceidae			-	-	-	-	25
	Malacostraca	Decapoda	Munididae		lobster krill	7	13	-	14	-
			Paguridae		hermit crabs	-	7	9	-	-
			Portunidae			29	20	18	-	-
		Euphausiacea	Euphausiidae		krill	-	-	-	14	25
		Isopoda			isopods	43	67	18	43	25
			Serolidae		Serolidae marine isopods	21	13	-	-	-
			Sphaeromatidae		marine pill bugs	36	53	18	43	25
	Arachnida	Oribatida			moss mites	7	-	-	-	-
		Trombidiformes	Halacaridae		meiobenthic mites	-	7	-	-	-
	Insecta	Coleoptera			beetles	21	20	-	-	-
			Dytiscidae		predaceous diving beetles	-	7	-	-	-
		Diptera			flies	-	-	-	14	-
Mollusca	Polyplacophora				sea cradles	7	13	-	-	-
	Bivalvia				bivalves	21	7	9	29	-
		Mytilida	Mytilidae		mussels	7	7	-	-	-
		Venerida				-	-	-	14	-
	Gastropoda		Nacellidae	*Nacella* sp.		57	33	36	-	-
		Neogastropoda	Cominellidae	*Pareuthria plumbea*	leaden whelk	29	7	18	-	-
		Fissurelloidea	Fissurellidae		keyhole limpets	-	7	-	-	-
		Trochida	Turbinidae		star snails	21	20	45	14	-
Echinodermata	Holothuroidea				sea cucumbers	-	7	-	-	-
	Asteroidea				sea stars	7	13	-	-	-
Chordata	Ascidiacea	Stolidobranchia	Molgulidae		ascidians	7	-	-	14	-

Sample sizes of Falkland Flightless Steamer Duck: females 16 (14), males 22 (15), chicks 11 (11). Sample sizes of Patagonian Crested Duck: adults 12 (7), chicks 4 (4). Sample sizes correspond to the number of DNA extractions from scat samples and, in brackets, to the number of successfully sequenced samples.

**Table 3:** Actinopterygii (fishes) consumed by Falkland Flightless Steamer Duck *Tachyeres brachydactyla* and Patagonian Crested Duck *Lophonetta specularoides*, and frequency of occurrence (% of samples).

Order	Family	Species	Common Name	Falkland Flightless Steamer Duck			Patagonian Crested Duck	
				Females	Males	Chicks	Adults	Chicks
			fishes	43	20	45	-	-
Clupeiformes	Clupeidae		herrings	-	7	-	-	-
	Engraulidae		anchovies	-	13	-	-	-
Myctophiformes	Myctophidae	*Gymnoscopelus braueri*	Brauer’s lanternfish	7	-	-	-	-
Scorpaeniformes	Agonidae		alligatorfishes	7	-	-	-	-
Perciformes				43	20	9	-	-
	Eleginopsidae	*Eleginops* sp.		7	-	-	-	-
		*Eleginops maclovinus*	Patagonian blennie	7	-	-	-	-
	Channichthyidae		crocodile icefishes	7	-	-	-	-
	Carangidae		Jacks	-	7	-	-	-
	Sparidae		sea bream fishes	29	20	9	-	-
	Scombridae	*Scomber* sp.	mackerels	-	7	-	-	-
	Nototheniidae	*Patagonotothen* sp.		38	20	9	-	-
		*Patagonotothen longipes*	Antarctic blennies	7	13	9	-	-
		*Patagonotothen ramsayi*	cod icefish	21	13	9	-	-
		*Patagonotothen sima*	humped rockcod	29	20	9	-	-

Sample sizes of Falkland Flightless Steamer Duck: females 16 (14), males 22 (15), chicks 11 (11). Sample sizes of Patagonian Crested Duck: adults 12 (7), chicks 4 (4). Sample sizes correspond to the number of DNA extractions from scat samples and, in brackets, to the number of successfully sequenced samples.

**Table 4:** Platyelminthes detected in Falkland Flightless Steamer Duck *Tachyeres brachydactyla* and Patagonian Crested Duck *Lophonetta specularoides*, and frequency of occurrence (% of samples).

Class	Order	Family	Common name	Falkland Flightless Steamer Duck			Patagonian Crested Duck	
				Females	Males	Chicks	Adults	Chicks
Cestoda			parasitic tapeworm	29	53	55	29	25
	Bothriocephalidea	Bothriocephalidae		7	-	-	-	-
	Cyclophyllidea			-	-	-	14	-
	Hymenolepididae			-	7	9	-	-
Trematoda			parasitic flatworms/flukes	64	20	18	-	25
	Plagiorchiida			29	7	18	-	25
		Notocotylidae		14	7	-	-	25

Sample sizes of Falkland Flightless Steamer Duck: females 16 (14), males 22 (15), chicks 11 (11). Sample sizes of Patagonian Crested Duck: adults 12 (7), chicks 4 (4). Sample sizes correspond to the number of DNA extractions from scat samples and, in brackets, to the number of successfully sequenced samples.

## Data Availability

All data supporting the conclusions of this article are available or cited within the article and its Supplementary Materials.

## Supplementary Materials

The following supporting information can be downloaded at https://www.mdpi.com/article/10.3390/genes14030731/s1, Figure S1. Falkland Flightless Steamer Duck (front) and Patagonian Crested Duck (back) sharing a beach environment on New Island. Note the female Falkland Flightless Steamer Duck defecating (front left); Figure S2. Female (right) and male (left) Falkland Flightless Steamer Duck from New Island; Figure S3. Falkland Flightless Steamer Duck parents and their chicks foraging in the vicinity of Protector Beach, New Island; Figure S4. Falkland Flightless Steamer Duck parents and their chicks resting on South Harbour Beach, New Island, where several of the scats were collected; Figure S5. Patagonian Crested Duck foraging on New Island; Figure S6. Patagonian Crested Duck adult and chicks foraging on Protector Beach, New Island; Figure S7. Patagonian Crested Duck adult foraging on Protector Beach, New Island; Figure S8: Diet composition using non-metric multidimensional scaling of molecular operational taxonomic units. Data correspond to Falkland Flightless Steamer Duck and Patagonian Crested Duck, foraging on the coast of New Island, Falkland/Malvinas Island, from October 2017 to February 2019. The names in the figure correspond to the family identity of the prey consumed. See also Table 2 and Table 3.
