# Supplementary material for: Using Next-Generation Sequencing to Disentangle the Diet and Incidence of Intestinal Parasites of Falkland Flightless Steamer Duck Tachyeres brachypterus and Patagonian Crested Duck Lophonetta specularioides Sharing a South Atlantic Island
Source: Genes (Basel). 2023 Mar 16;14(3):731. doi: 10.3390/genes14030731 (PMC10048246; doi:10.3390/genes14030731)
Supplement: Supplementary file 1 [file genes-14-00731-s001.zip › NGS_&_diet_Falkland_ducks_Supplementary Materials_1_Figs_S1_to_S7.pdf]

Supplementary Materials

Using next generation sequencing to disentangle the diet and incidence of intestinal parasites of Falkland Flightless Steamer Duck *Tachyeres brachypterus* and Patagonian Crested Ducks *Lophonetta specularioides* sharing a South Atlantic island

Juan F. Masello [1,*], Yvonne R. Schumm [1], Sven Griep [2] and Petra Quillfeldt [1]

[1] Department of Animal Ecology & Systematics, Justus Liebig University Giessen, Heinrich-Buff-Ring 26, D-35392 Giessen, Germany;
[2] Institute for Bioinformatics & Systems, Biology Justus Liebig University Giessen, Heinrich-Buff-Ring 58, D-35392 Giessen, Germany;
* Correspondence: juan.masello@gmx.de

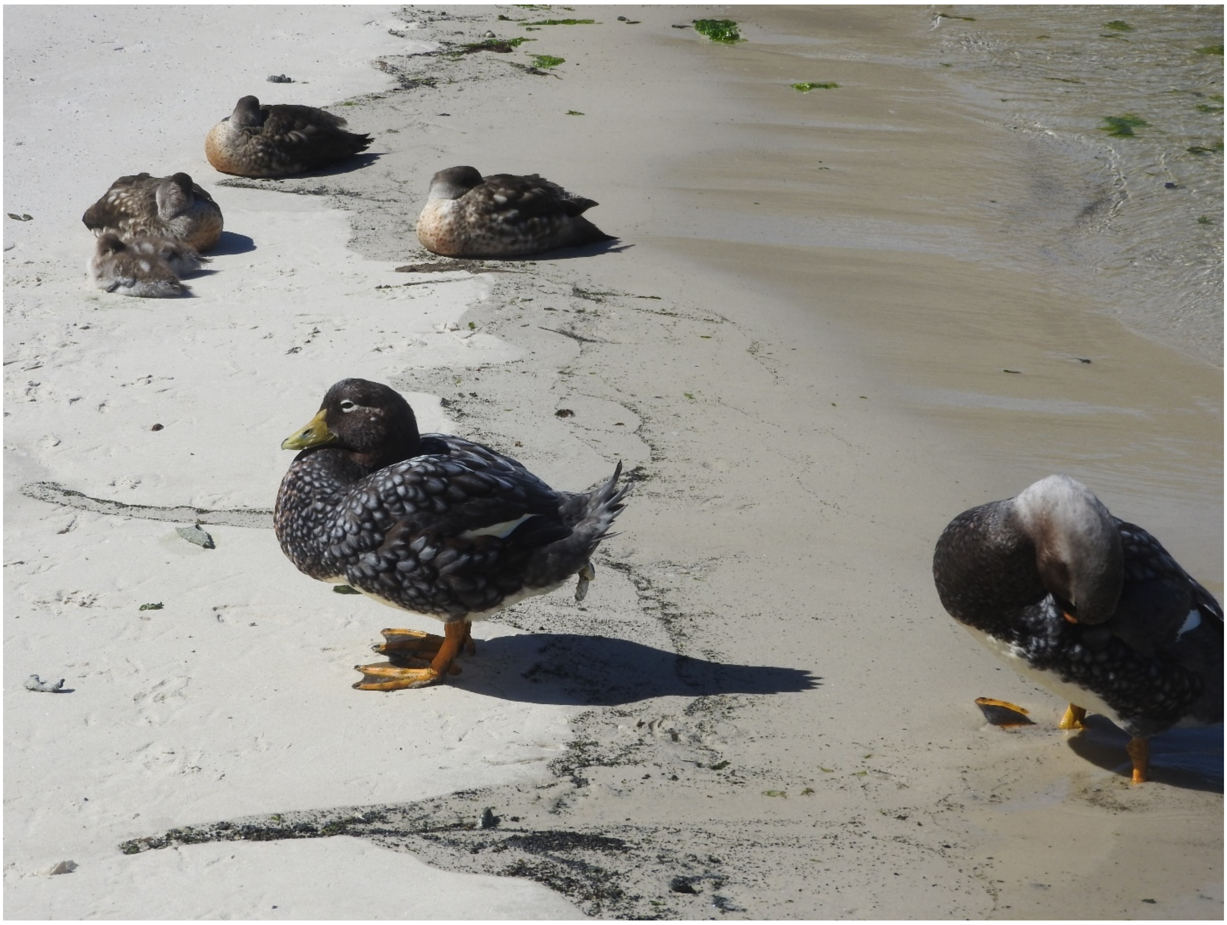

Figure S1. Falkland Flightless Steamer Duck *Tachyeres brachypterus* (front) and Patagonian Crested Ducks *Lophonetta specularioides* (back) sharing a beach environment on New Island. Note the female Falkland Flightless Steamer Duck defecating (front left). Photo credit: Petra Quillfeldt

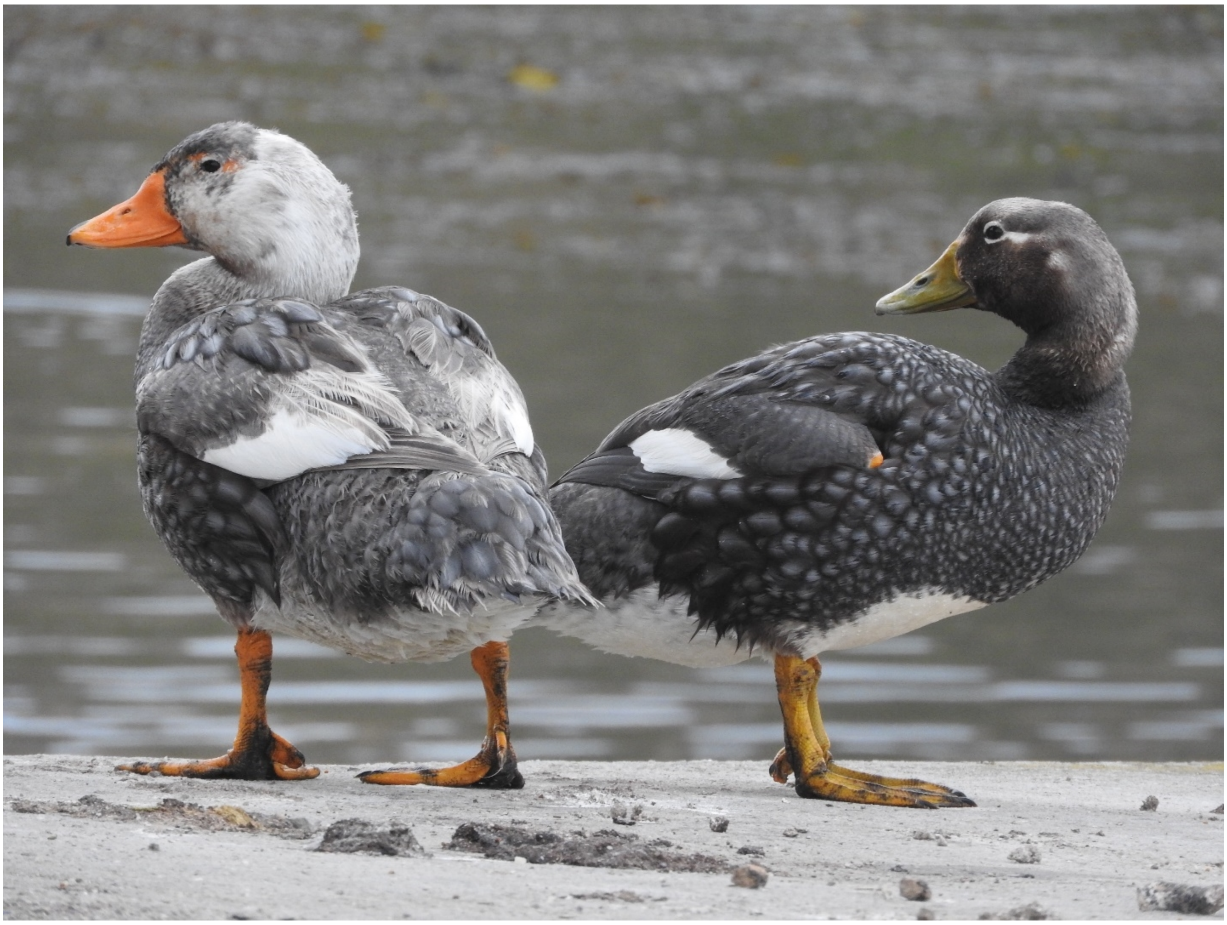

Figure S2. Female (right) and male (left) Falkland Flightless Steamer Duck *Tachyeres brachydactyla* from New Island. Photo credit: Petra Quillfeldt

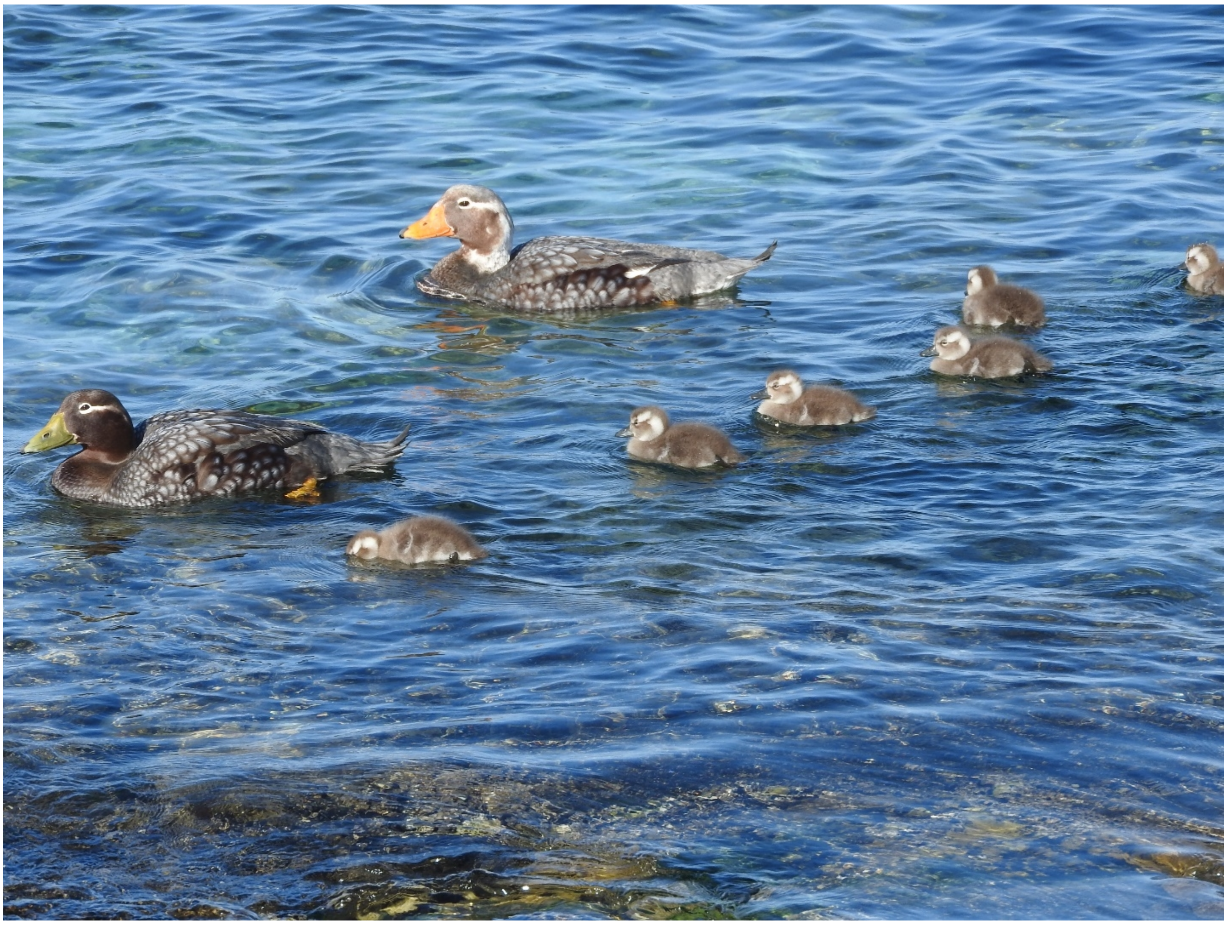

Figure S3. Falkland Flightless Steamer Duck *Tachyeres brachydactyla* parents and their chicks foraging in the vicinity of Protector Beach, New Island. Photo credit: Petra Quillfeldt

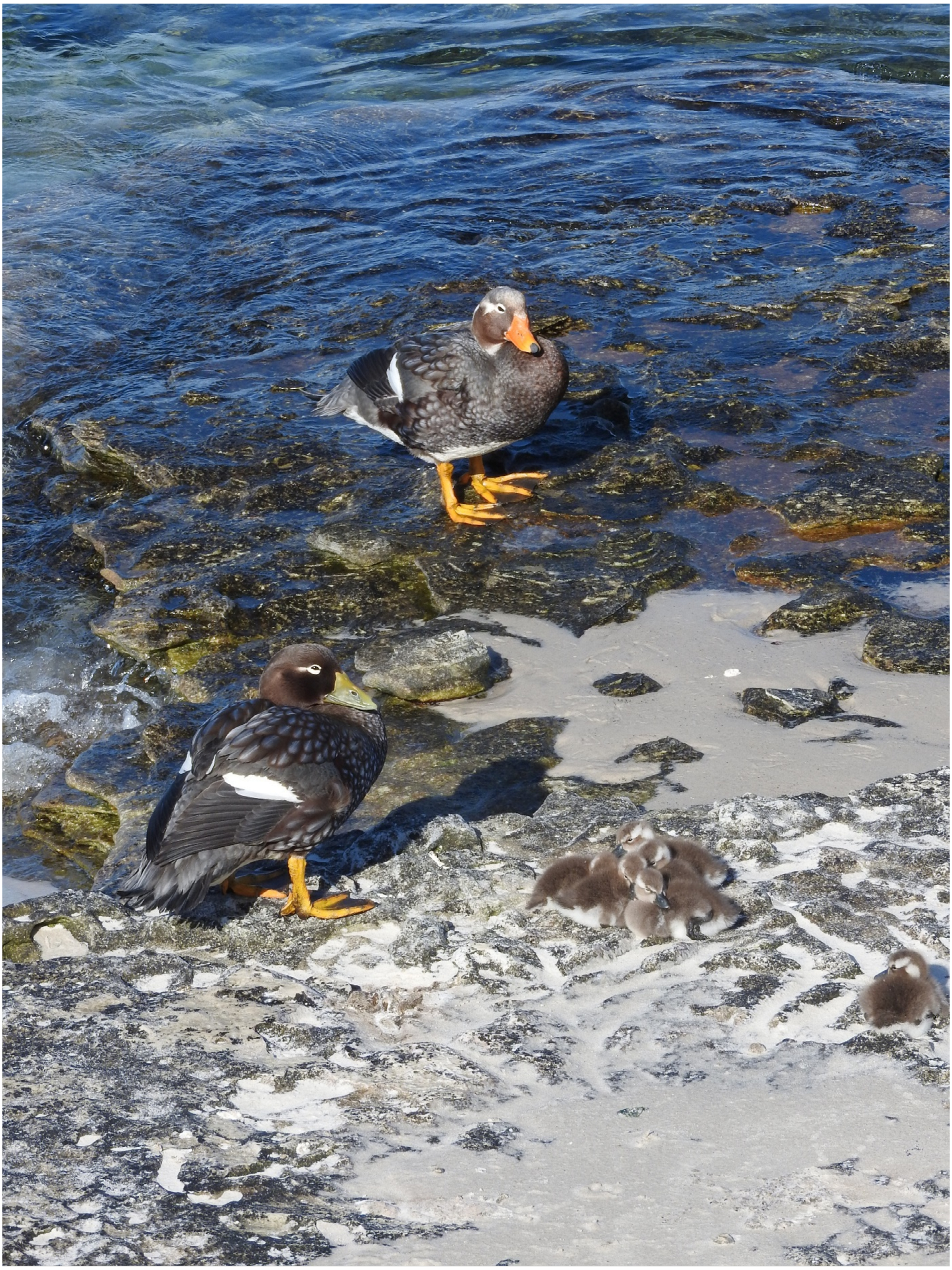

Figure S4. Falkland Flightless Steamer Duck *Tachyeres brachydactyla* parents and their chicks resting on South Harbour Beach, New Island, where several of the scats were collected. Photo credit: Petra Quillfeldt

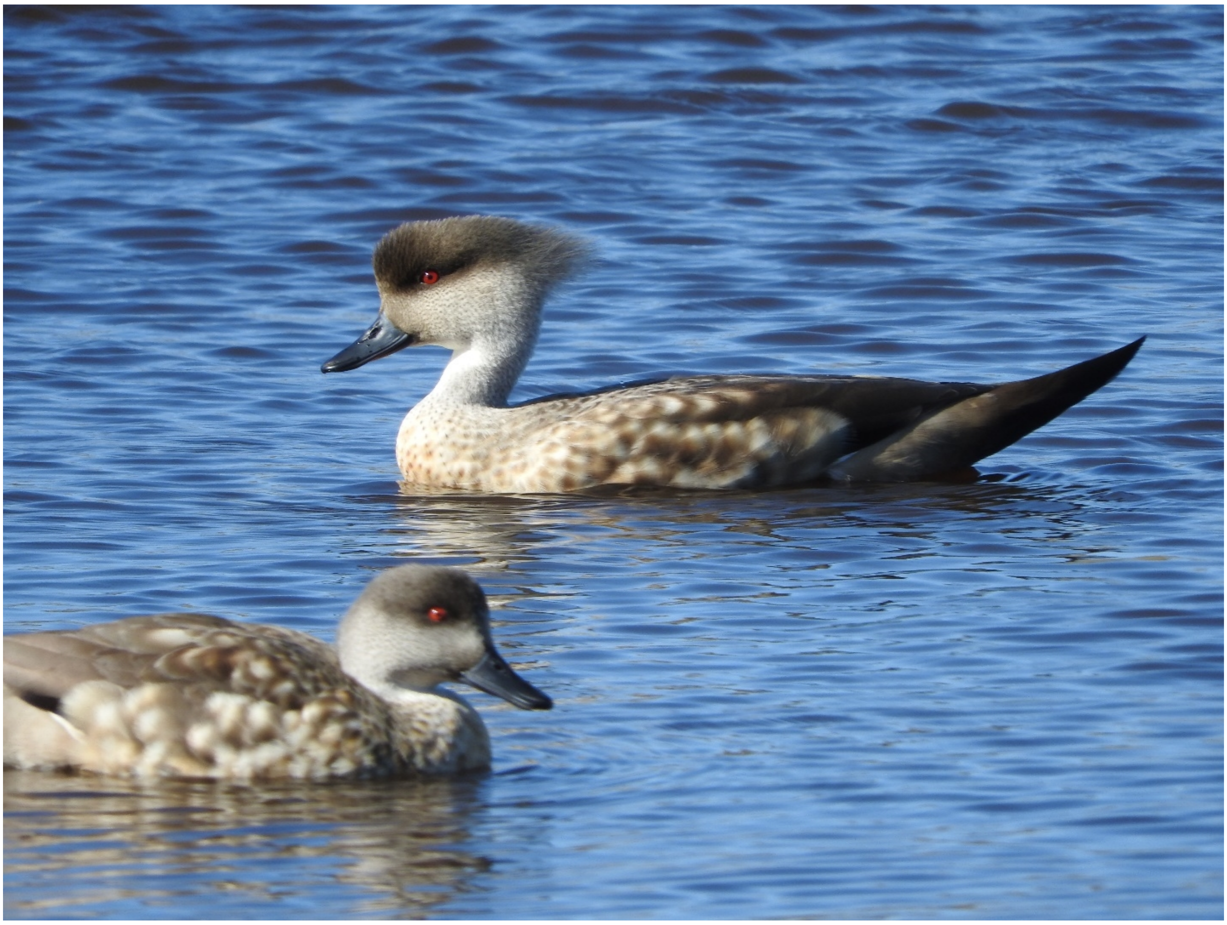

Figure S5. Patagonian Crested Duck *Lophonetta specularoides* foraging on New Island. Photo credit: Petra Quillfeldt

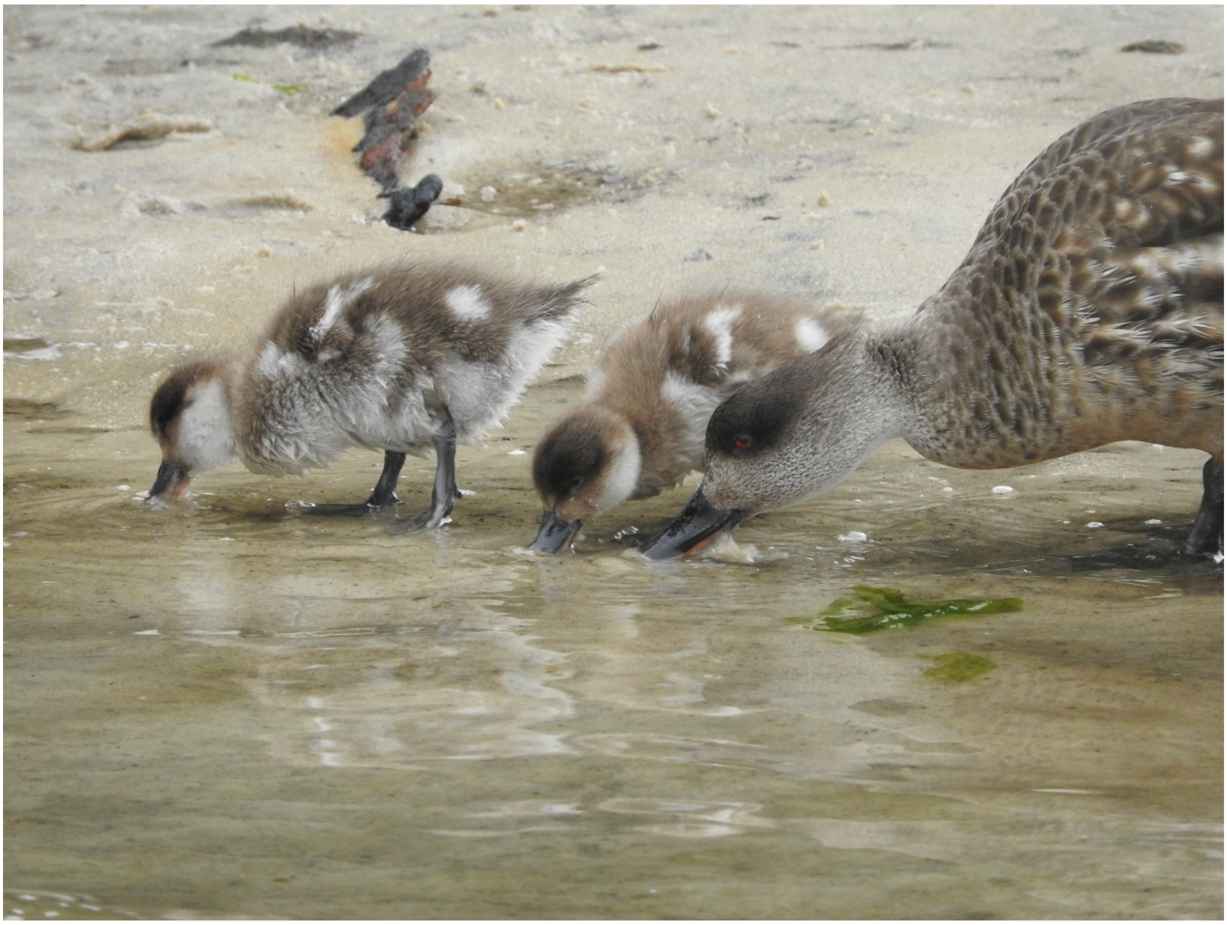

Figure S6. Patagonian Crested Duck *Lophonetta specularoides* adult and chicks foraging on Protector Beach, New Island. Photo credit: Petra Quillfeldt

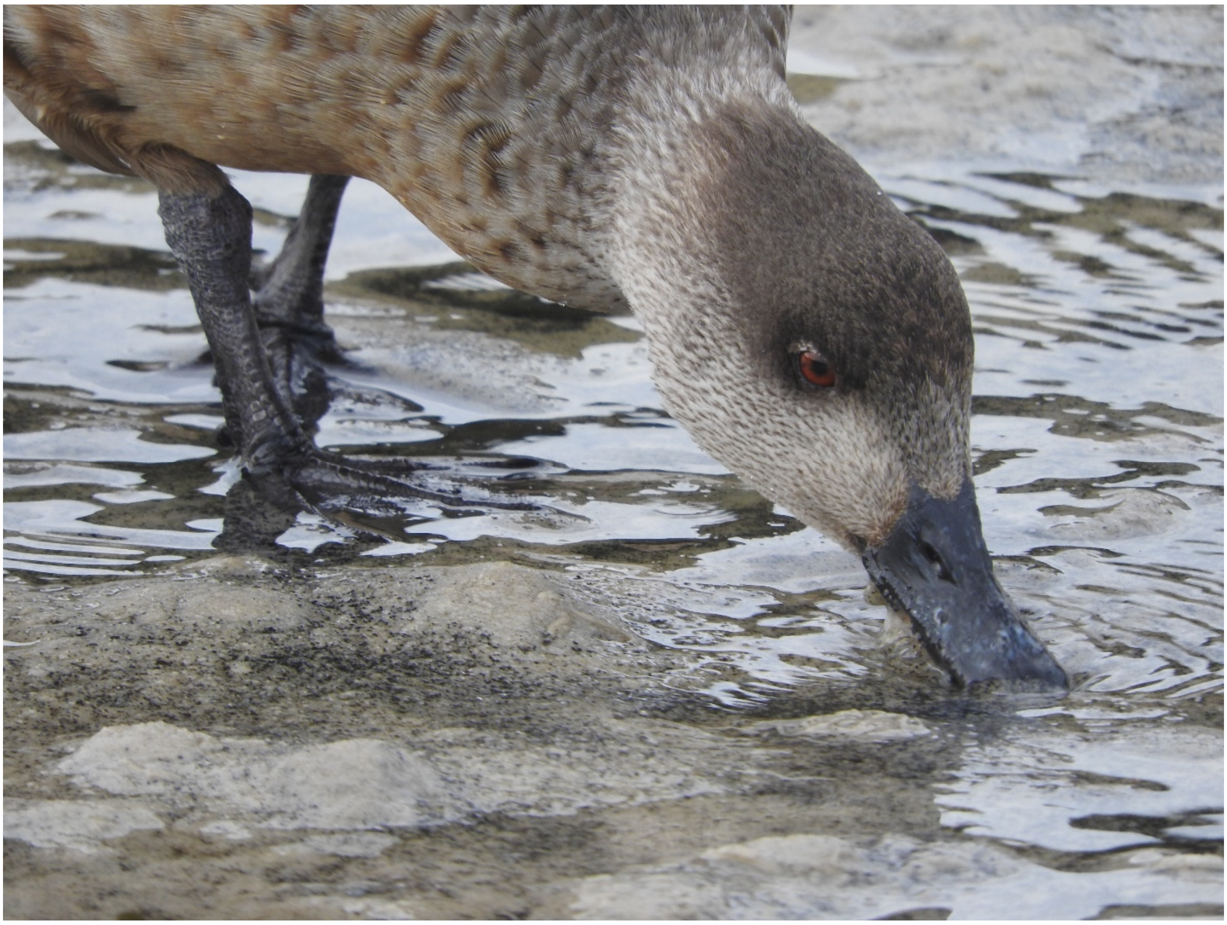

Figure S7. Patagonian Crested Duck *Lophonetta specularoides* adult foraging on Protector Beach, New Island. Photo credit: Petra Quillfeldt
