# Supplementary figures and images for: Using Next-Generation Sequencing to Disentangle the Diet and Incidence of Intestinal Parasites of Falkland Flightless Steamer Duck Tachyeres brachypterus and Patagonian Crested Duck Lophonetta specularioides Sharing a South Atlantic Island
Source: Genes (Basel). 2023 Mar 16;14(3):731. doi: 10.3390/genes14030731 (PMC10048246; doi:10.3390/genes14030731)

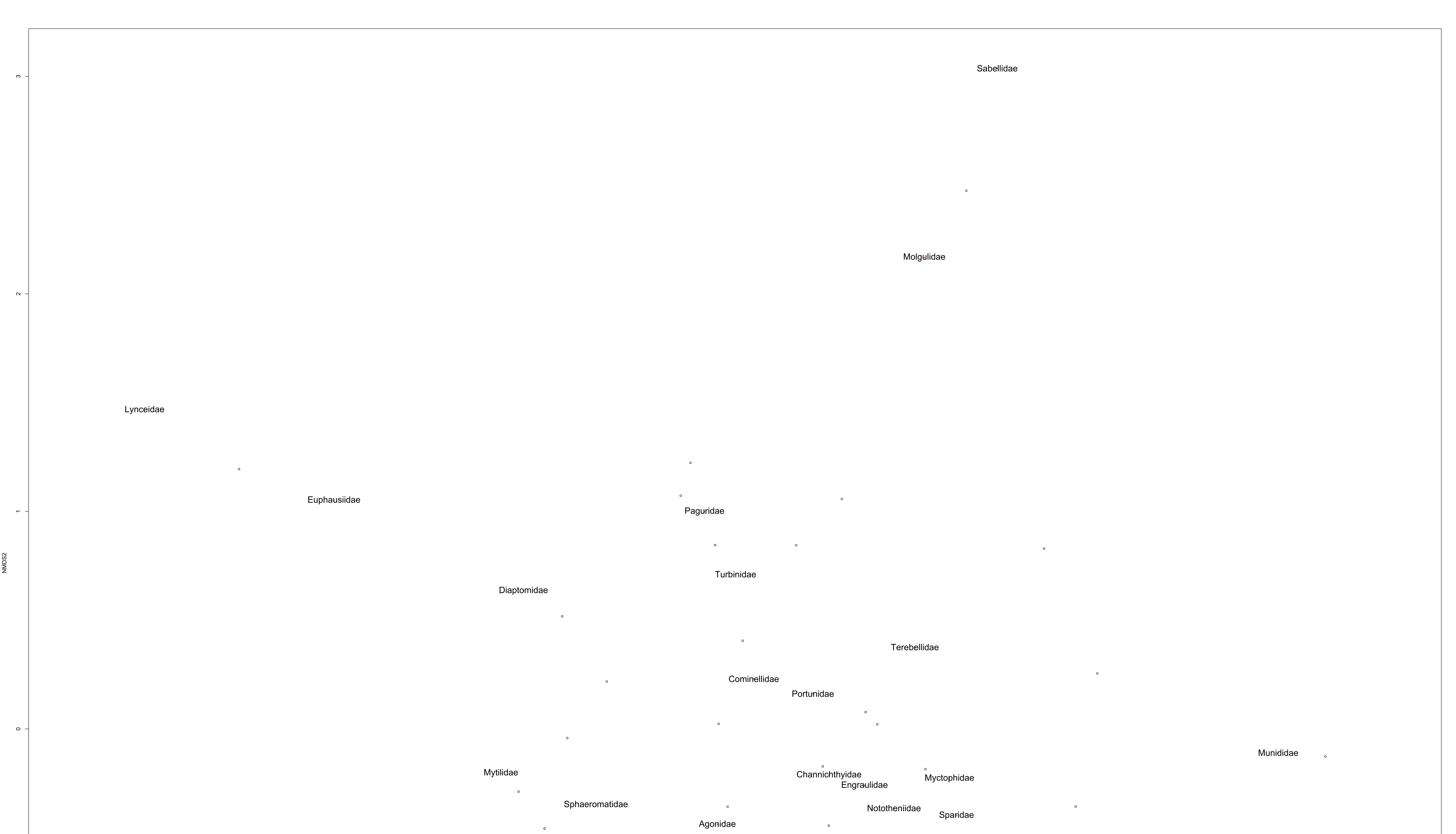

Supplement: Supplementary file 1 [file genes-14-00731-s001.zip › NGS_&_diet_Falkland_ducks_Supplementary Materials_2_Figure_S8.pdf]
